# Syphilis in the economic center of South China: results from a real-time, web-based surveillance program

**DOI:** 10.1186/s12879-015-1072-z

**Published:** 2015-08-08

**Authors:** Wangjian Zhang, Zhicheng Du, Shaokai Tang, Pi Guo, Xingdong Ye, Yuantao Hao

**Affiliations:** Department of Medical Statistics and Epidemiology & Health Information Research Center & Guangdong Key Laboratory of Medicine, School of Public Health, Sun Yat-sen University, Guangzhou, 510080 Guangdong Province China; Guangzhou Institute of Dermatology, Guangzhou, 510095 Guangdong Province China

## Abstract

**Background:**

Guangzhou is the economic center of South China, which is currently suffering an insidious re-emergence of syphilis. Syphilis epidemic in this area is a matter of serious concern, because of the special economic position of Guangzhou and its large migrant population. Therefore, a comprehensive analysis of surveillance data is needed to provide further information for developing targeted control programs.

**Method:**

Case-based surveillance data obtained from a real-time, web-based system were analyzed. A hierarchical clustering method was applied to classify the 12 districts of Guangzhou into several epidemiological regions. The district-level annual incidence and clustering results were displayed on the same map to show the spatial patterns of syphilis in Guangzhou.

**Results:**

A total of 60,178 syphilis cases were reported during the period from 2005 to 2013, among which primary/secondary syphilis accounted for 15,864 cases (26.36 %), latent syphilis for 41,078 cases (68.26 %) and congenital syphilis for 2,090 cases (3.47 %). Moreover, primary/secondary syphilis burden slightly decreased from 17.5-18.0 cases per 100,000 people in the first years to 10.6 cases per 100,000 in 2013, with latent syphilis largely increasing from 18.5 cases per 100,000 to 43.4 cases per 100,000. Districts of Guangzhou could be classified into 3 epidemiological regions according to the syphilis burden over the last 3 years of the study period.

**Conclusions:**

The burden of primary/secondary syphilis appears to be decreasing in recent years, whereas that of latent syphilis is increasing. Given the epidemiological features and the annual changes found in this study, it is suggested that future control programs should be more population-specific and spatially targeted.

**Electronic supplementary material:**

The online version of this article (doi:10.1186/s12879-015-1072-z) contains supplementary material, which is available to authorized users.

## Background

Syphilis is a sexually transmitted infection (STI) that causes cutaneous manifestations such as genital ulcers, and a variety of complications including neurologic, renal, gastrointestinal, and hepatic diseases [1]. Previous work has provided evidence of the effects of syphilis on abortion, congenital defects, severe neonatal diseases, and even the spread of HIV [2–4]. Syphilis control should therefore be regarded as a public health priority.

After being virtually eradicated in the 1960s, syphilis has made a re-emergence in China since the initiation of economic and social reforms [5]. The country has seen a precipitous increase in reported syphilis cases over the past two decades, particularly in Guangdong Province, the largest province in South China [6, 7]. The single province of Guangdong reported more syphilis cases than all of the countries in the European Union in 2008 [8]. This situation has not improved in more recent years [9].

Guangzhou is one of the cities most affected by syphilis within Guangdong province. During the period of 2000 to 2011, syphilis burden in this area was tripled that of the national average [10]. Given its specific position as the capital city of Guangdong Province and the de facto economic center of South China, as well as the large population involved (over 12 million in 2013), syphilis epidemics in Guangzhou are of great concern [10, 11].

Analysis of surveillance data is believed to guide control strategies in Guangzhou. However, there are few studies of this possibility. In addition, previous work is limited by being low resolution, non-spatial, or even cross-sectional, because information collected by the reporting card-based surveillance system before 2005 was quite limited [10, 11].

This research provides a comprehensive picture of epidemiological features of syphilis and associated shifts over years in Guangzhou, based on surveillance data collected by a real-time, web-based system between 2005 and 2013. To make the analysis more vivid and meaningful, distribution patterns of syphilis in Guangzhou was analyzed by different disease stages, age groups, and spatial locations. The primary objective of this analysis was to identify demographic and spatial subpopulations that may have a higher syphilis burden, to inform further targeted control programs.

## Method

### Ethics statement

This study was based on official syphilis surveillance data in Guangzhou. Analyses were conducted at the aggregate level and no confidential information was involved. The research study protocol was approved by the Institutional Review Board of Guangzhou Institute of Dermatology.

### Surveillance data of syphilis and population data

Case-based syphilis surveillance data collected between 2005 and 2013 were obtained from the Guangzhou Institute of Dermatology. According to the Diagnostic criteria for Syphilis of the National STD Control Center [12], primary and secondary syphilis cases were determined by positive treponemal and non-treponemal tests in addition to characteristic clinical findings and sexual risk history, whereas asymptomatic individuals with positive test results were diagnosed as latent syphilis cases. Patients infected with treponema pallidium for more than 2 years were diagnosed as tertiary syphilis cases, including benign cutaneous syphilis cases, neurosyphilis cases, and cardiovascular syphilis cases. Diagnosis of congenital syphilis cases is more complex, as specified in national criteria [12]. A case was reported to the case-based surveillance system (CBSS) only when he or she was first diagnosed as having syphilis after being infected with treponema pallidium (TP). Information on age, gender, onset time, stage of syphilis, etc., was entered into the CBSS.

Primary/secondary and latent syphilis cases were the focus of this study. The former are early-stage syphilis cases more likely related to recent exposure and transmission, whereas the latter reflect the extent of routine syphilis screening in the area. Congenital syphilis cases were also targeted in this work, since such cases represent good indicators of the coverage and efficiency of pre-marital/pre-natal screening and intervention.

District-level annual population data for the study period were obtained from Guangzhou Statistical Yearbooks.

### Statistical analysis

Different measures including the reported case number, incidence, proportion, and male-to-female ratio were calculated for the three kinds of syphilis cases from 2005 to 2013. Variables including marriage status, education and the most probable route of acquisition were calculated for primary/secondary and latent syphilis cases in this time frame, given that congenital syphilis cases tended to be concentrated in children, with a definite acquisition route. Information on marriage status, education and the most probable route of acquisition was only available from 2008 to 2013.

Cases were then aggregated to each of the 12 districts of Guangzhou according to the ZIP code of the reported current addresses of individuals. Spatial patterns of syphilis were studied at the district level. The faceting strategy in *ggplot2* package was used to produce multiple bar charts displaying the stage-and-district-specific cases and incidence. Faceting is a mechanism for automatically laying out multiple plots on a page. It splits the data into subsets, and then plots each subset into a different panel on the page [13]. Hierarchical clustering with Ward’s minimum variance method in *cluster* package was applied to classify these districts into epidemiological regions according to their syphilis burdens. Clustering is a major method to group districts with similar disease burdens and provide information on which districts are suffering similar syphilis epidemics and should be classified into the same epidemiological region [14, 15]. Surveillance data for the last three years was used for these district-level analyses.

Result mapping was conducted using ArcGIS10.0. All the other analyses were accomplished using R3.0.2.

## Results

1. Cases and incidence of syphilis

**Fig. 1:**
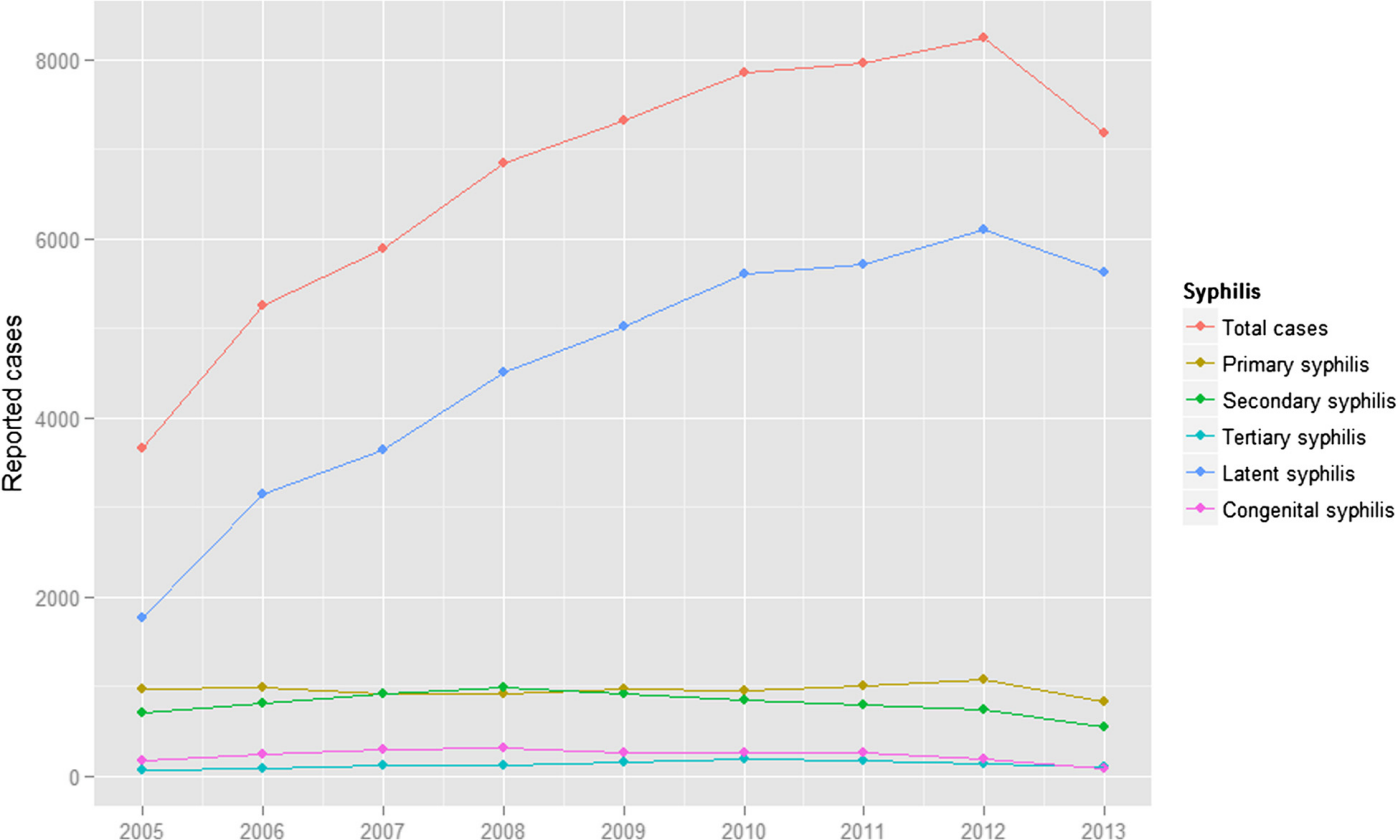
Reported syphilis cases in Guangzhou from 2005 to 2013

A total of 60,178 cases of syphilis infections were reported to the CBSS during the past nine years, from 2005 to 2013 (Fig. 1). During the study period, primary/secondary syphilis cases accounted for 15,864 (26.36 %) of the total reported cases, whereas the figures for latent and congenital syphilis cases were 41,078 (68.26 %) and 2,090 (3.47 %), respectively.

Primary/secondary syphilis burden decreased slightly, from 17.5-18.0 cases per 100,000 people in earlier years of the study period to 10.6 cases per 100,000 in 2013. Latent syphilis cases represented the largest share of total reported cases throughout the study period. An apparent increase (from 18.5 cases per 100,000 to 47.5 cases per 100,000) in latent syphilis burden was seen from 2005 to 2012. The figure in 2013 was 43.4 per 100,000 individuals. Congenital syphilis burden increased slightly from 1.7 cases per 100,000 in 2005 to 2.9 cases per 100,000 in 2007, and then decreased annually to 0.7 cases per 100,000 in 2013 (Additional file 1: Figure S1).

2) Characteristics of syphilis cases

**Fig. 2:**
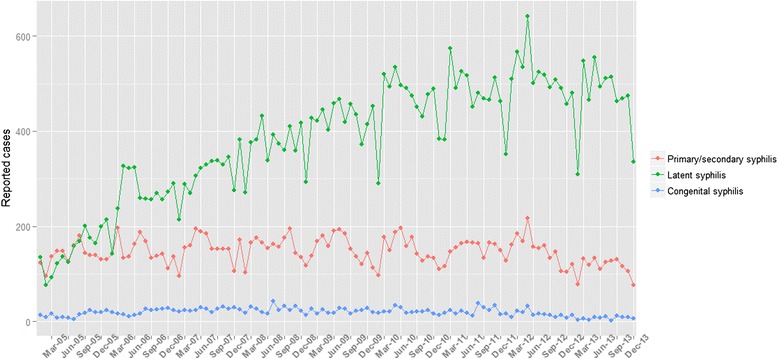
Time series plot for reported syphilis cases in Guangzhou from 2005 to 2013

Periodicity within the year was apparent for primary/secondary, as well as latent syphilis cases (Fig. 2). Winter months seem to be associated with fewer reports than other months of the year, with the exception of more sporadic reports of congenital syphilis cases.

**Fig. 3:**
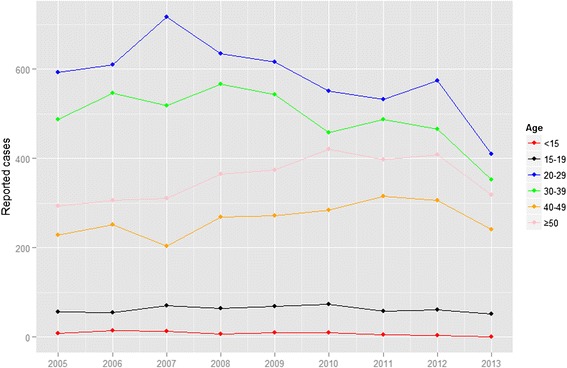
Age-specific primary/secondary syphilis cases in Guangzhou from 2005 to 2013

Primary/secondary and latent syphilis cases more than 20 years of age showed a temporal shift (Fig. 3, Additional file 2: Figure S2). Among primary/secondary syphilis cases, people aged 20–39 years largely decreased over the assessed time frame, whereas there was an increase in individuals over 50 years of age. Moreover, all age groups above 20 years of age showed increases among latent syphilis cases. Almost all (99.1 %) congenital syphilis cases were concentrated in children under 15 years old.

The male-to-female ratio of latent syphilis cases was approximately 1.0 and was lower than the figure for other two types of syphilis cases. In addition, the figure for latent syphilis cases decreased from 2011 onwards, whereas the figures for other two types of syphilis increased annually during the same period.

Data regarding marriage status, education and the most probable acquisition route was also collected as part of CBSS. These characteristics were similar for primary/secondary and latent syphilis cases, among which married individuals and individuals with middle school, or high school education represented a large proportion (Table 1). Moreover, approximately half of primary/secondary and latent syphilis cases were originally infected through heterosexual behaviors (Table 1).

**Table 1:** Some features of primary/secondary and latent syphilis cases, median (IQR)

	Measurements	Primary/secondary syphilis (%)	Latent syphilis (%)
Marriage	Married	74.59 (73.65,73.95)	83.61 (82.77,83.67)
	Unmarried	23.19 (22.69,23.66)	12.75 (12.49,12.71)
	Divorced/widowed	2.51 (2.01,2.39)	3.95 (3.41,3.62)
Education	Collage or above	15.24 (14.96,15.48)	12.2 (11.79,12.28)
	High	28.62 (27.12,27.59)	28.99 (28.16,28.26)
	Middle	40.93 (39.96,41.49)	36.68 (36.05,37.84)
	Primary or below	15.38 (14.51,15.44)	21.55 (21.05,21.63)
Transmitted	Homosexual	1.04 (0.54,1.47)	0.26 (0.13,0.33)
	Drug user	0.53 (0.39,0.51)	2.52 (2.18,2.56)
	Heterosexual	44.13 (41.17,44.61)	39.21 (38.13,40.01)
	Blood related	0.45 (0.33,0.43)	0.67 (0.63,0.70)
	Other routes	54.03 (48.05,52.98)	57.07 (54.84,56.39)

3) Spatial analysis

**Fig. 4:**
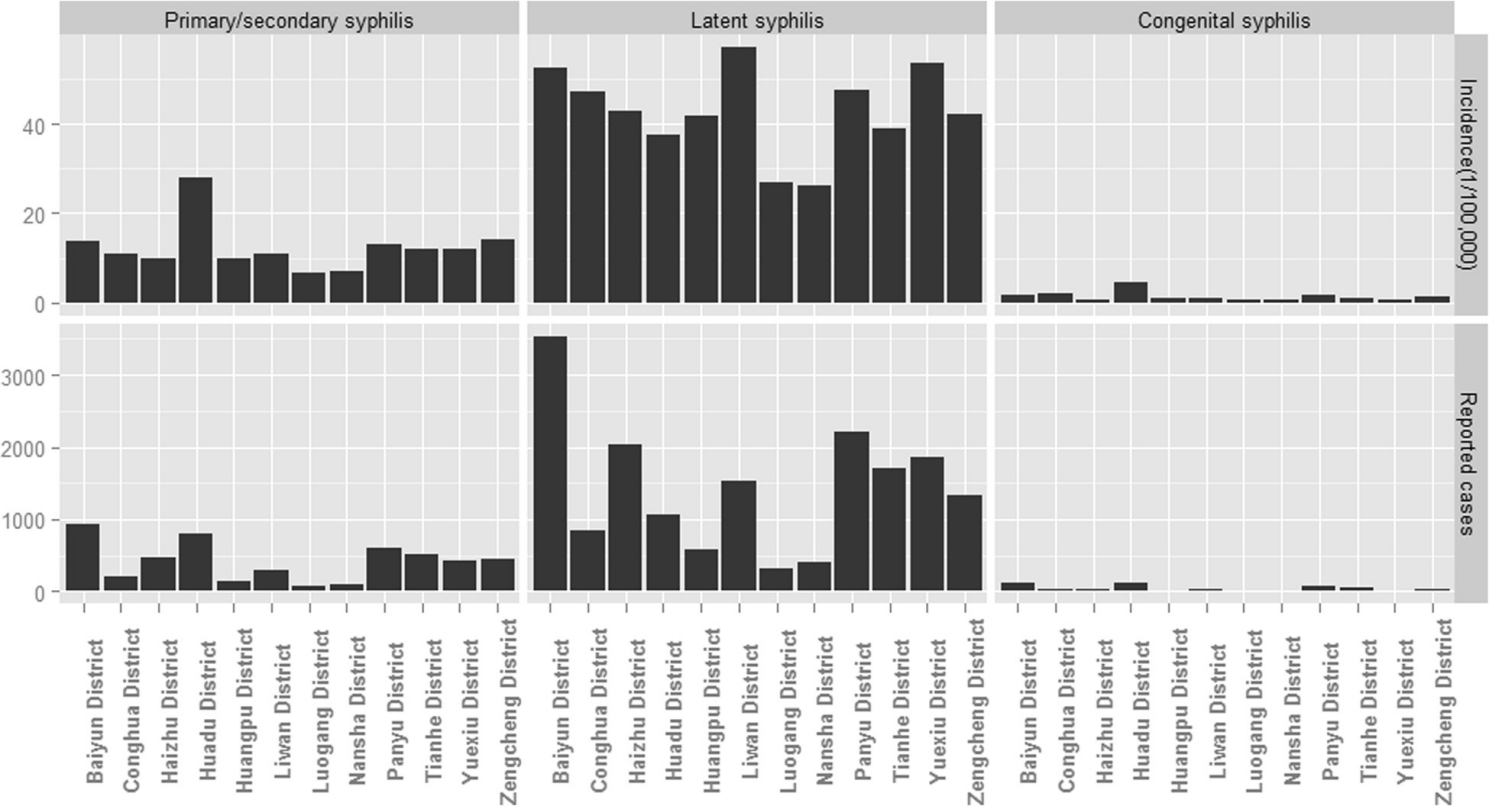
Syphilis distribution pattern at the county level in Guangzhou from 2011 to 2013

According to the results of district-level analyses (Fig. 4), in the last 3 years of the study period, Baiyun District (926 cases) reported the highest number of primary/secondary syphilis cases, followed by Huadu District (799 cases) and Panyu District (606 cases). Baiyun District (3,540 cases) also reported the highest number of latent syphilis cases, followed by Panyu District (2,216 cases) and Haizhu District (2,024 cases). More congenital syphilis cases were reported in Huadu District (126 cases), Baiyun District (116 cases) and Panyu District (77 cases) than in other districts.

When population size was taken into consideration, the Huadu Distinct had heavier primary/secondary and congenital syphilis burdens, with a rate of 27.9 cases per 100,000 for the former and 4.4 cases per 100,000 for the latter. Other districts, including Liwan, Yuexiu and Baiyun, also suffered latent syphilis burdens of over 50.0 cases per 100,000.

**Fig. 5:**
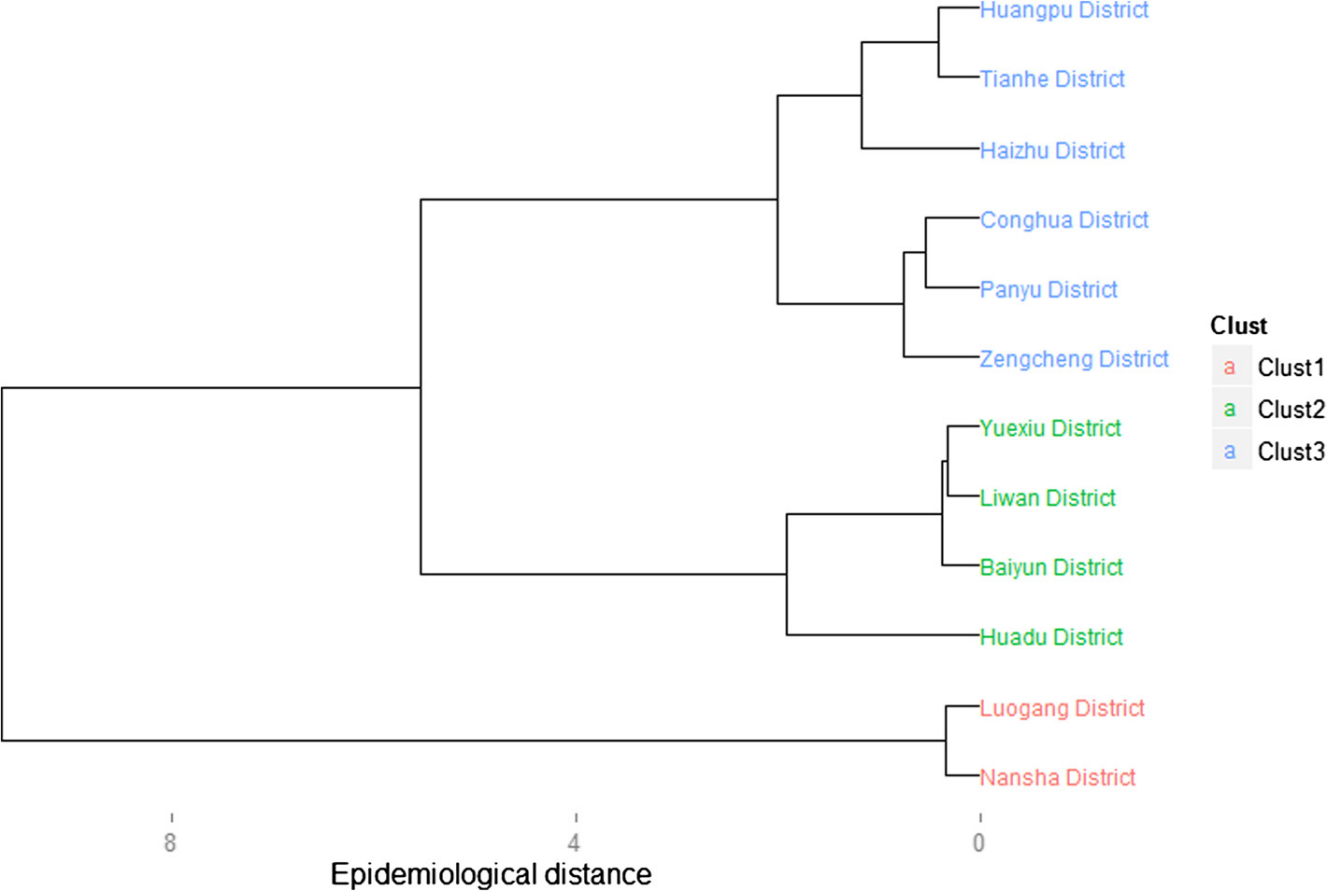
Hierarchical clustering based on total syphilis burden from 2011 to 2013

**Fig. 6:**
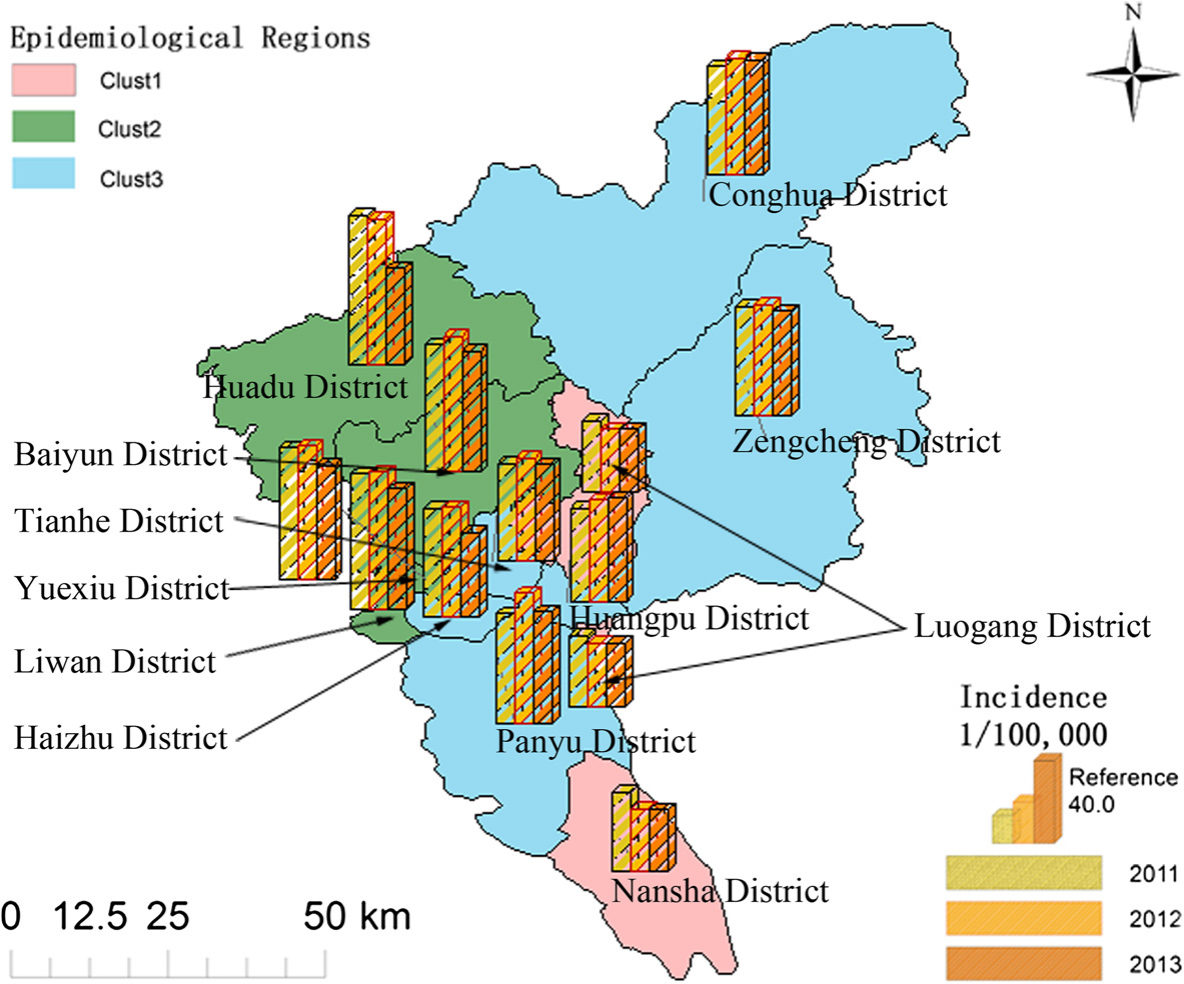
Mapping of district-level annually syphilis incidence and hierarchical clustering results, Guangzhou, 2011 to 2013

Lastly, the 12 districts of Guangzhou were classified into three epidemiological regions (Fig. 5). Among these regions (Fig. 6), Region 2, consisting of 4 districts of Yuexiu, Liwan, Baiyun and Huadu, had the highest burden of syphilis (66.13 cases per 100,000), whereas Region 1, consisting of Luogang and Nansha, had the lowest burden of syphilis (34.05 cases per 100,000). All the other districts were classified as Region 3 with an average incidence of 56.26 cases per 100,000.

## Discussion

In addition to the special economic status of Guangzhou in South China, syphilis epidemics in this area are of particular concern due to the large number and proportion of migrants among its population. It has been reported that migrants, with an estimated population of 8.37 million in 2013, had come to outnumber the registered resident population of Guangzhou, consisting of more than half of the total population in Guangzhou [16]. This large population of migrants contributed to the social and economic development of the area but simultaneously laid the foundation for increases in sexual transmitted infections (STIs) [17]. Far from being confined locally, syphilis epidemics in Guangzhou could have national or even international effects [18, 19]. Though sporadic previous studies have reported syphilis epidemics in Guangzhou [10], this is the first comprehensive description of epidemiological features and associated changes over time, based completely on real-time, web-based surveillance data.

Our results demonstrated that primary/secondary and congenital syphilis burden had largely decreased over recent years. This finding was expected. During the past nine years from 2005 to 2013, a series of interventions including training programs for entertainment venue owners and female sex workers (FSWs), to promote the usage of condoms [20], and prevention and control programs targeting mother-to-child syphilis transmission [21], were implemented to curb the growth of STIs. Latent syphilis burden has increased annually, which might be the result of growth in screening programs [22, 23]. Implementation of interventions also appears to have reduced the transformation of latent syphilis into primary/secondary syphilis.

There is periodicity in reported syphilis cases, as shown by the time series plot (Fig. 2), with fewer cases being reported during the winter months. Periodicity has been documented in previous syphilis researches. [24, 25] The reasons for periodicity might be that summer months lead to increased sexual activity due to increased travel and leisure activities. In addition, temperature is another presumed risk factor for syphilis burden as it influences hormone levels and leads to increased unsafe sexual behaviors, resulting in a higher possibility of getting infected with syphilis [26].

Age and sex are also presumed risk factors for syphilis. Sexual behaviors vary between different age and sex groups, which particularly affects the primary/secondary syphilis burden. Results of this study indicated that, cases of infection in sexually active populations have decreased in general, whereas there has been an annual increase of infections in people aged 50 years or older. The former decrease is expected, given that sexually active people tended to be the main target population for interventions, whereas the latter increase is somewhat unexpected. However, this finding does reinforce a series of reports released by local media in Guangzhou [27]. Most of those reports interpreted this pattern as the result of an increasing number of older people purchasing sex and called for more attention to sexual health issues in older populations [28, 29].

Male-to-female ratio is an index that comprehensively reflects between-sex disparities in population size, sexual behaviors, screening program attendance rates among others. More male than female cases were identified in the present study, although more detailed data are needed to further understand this pattern.

Married individuals, middle-school or high-school educated individuals and heterosexually infected individuals represented a large proportion of reported syphilis cases. Although this finding might provide limited information about the risk for specific population because of the absence of background data, it does provide us with valuable information about how interventions should be implemented. For instance, the findings of this study imply that sexual awareness campaigns and condom use promotion programs should be targeted at less educated people and FSWs, as well as married individuals.

Spatial analysis is an effective method for exploring the spatial patterns of infectious diseases. Simultaneously displaying the district-level annual incidence and clustering results on a map helps to identify the districts, or areas, that are suffering similar syphilis epidemics, and thereby suggest where and how interventions should be implemented based on the severity of illness burden. For instance, in Guangzhou, the syphilis burdens for the four districts in epidemiological Region 2 were similar, and were heavier than burdens for other districts, suggesting that this region requires more intervention resources. It is suggested that the methods used in this study could be adopted for analyzing patterns of other disease in different regions.

There are several limitations of this analysis of surveillance data. First, underreporting of STIs is commonplace [30, 31]. This research provides no exception, although real-time, web-based data collection could work to mitigate this limitation, to some extent. Second, it is difficult to accurately determine onset times, particularly in the case of latent syphilis cases. To minimize this shortcoming, the analysis in this study was mainly conducted at the annual level. Third, incidence is a good indicator of risk and is helpful when comparing the burden of syphilis to other areas. However, age-specific, sex-specific or other attribute specific incidences were not reported in this study, because the corresponding background varies substantially between different areas and remains unknown. To make results more meaningful, the absolute case number and proportion, which are good indicators of what subpopulations should be targeted for greater intervention and resource allocation, were reported. Stage-specific incidence was calculated with the total population as the denominator. Last, the impacts of individual characteristics including marriage status, education, and the most probable route of acquisition on syphilis was not assessed in the current study because of the lack of information for those not infected. Future case–control studies could be conducted to complement this kind of surveillance data-based studies.

## Conclusion

The burden of primary/secondary and congenital syphilis has largely decreased in Guangzhou in recent years, whereas the burden of latent syphilis has increased. Risk for older people aged 50 years and older has increased annually. According to syphilis burden statistics for the last three years, the twelve districts in Guangzhou City could be classified into three epidemiological regions, among which Region 2 is suffering a heavier syphilis burden, whereas Region 1 is comparatively better. Future control programs should be more population-specific and spatially targeted.

## Additional files

Additional file 1: Figure S1. Stage-specific syphilis incidence in Guangzhou from 2005 to 2013. (TIFF 1713 kb) Additional file 2: Figure S2. Age-specific latent syphilis cases in Guangzhou from 2005 to 2013. (TIFF 1471 kb)

## Abbreviations

CDC: Center for Disease Control and Prevention
STI/STD: Sexually transmitted infections/disease
CBSS: Case-based surveillance system
FSW: Female sex worker
IQR: Interquartile range
