# Supplementary figures and images for: Syphilis in the economic center of South China: results from a real-time, web-based surveillance program
Source: BMC Infect Dis. 2015 Aug 8;15:318. doi: 10.1186/s12879-015-1072-z (PMC4545813; doi:10.1186/s12879-015-1072-z)

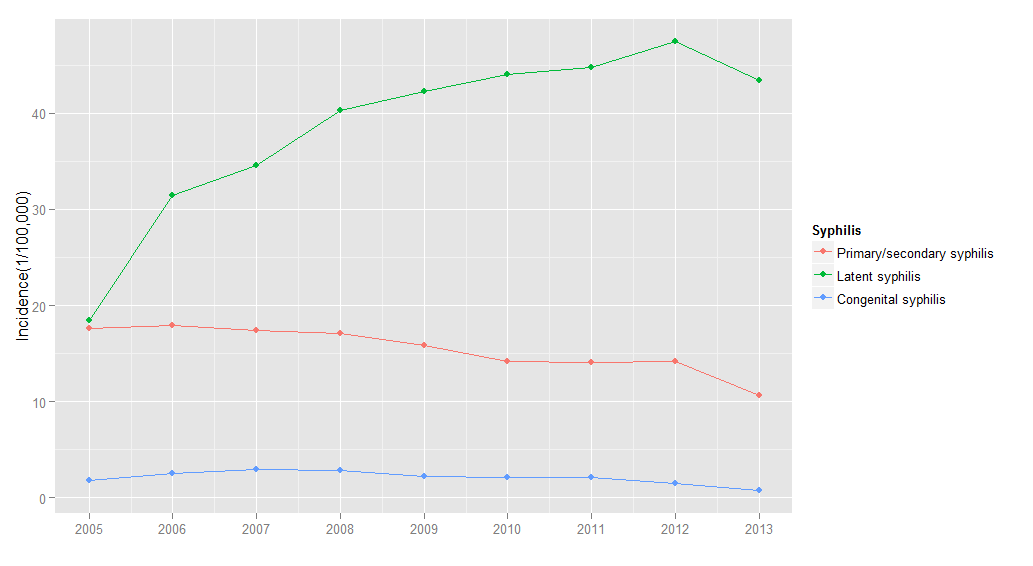

Supplement: Additional file 1: Figure S1. — Stage-specific syphilis incidence in Guangzhou from 2005 to 2013. (TIFF 1713 kb) [file 12879_2015_1072_MOESM1_ESM.tiff]

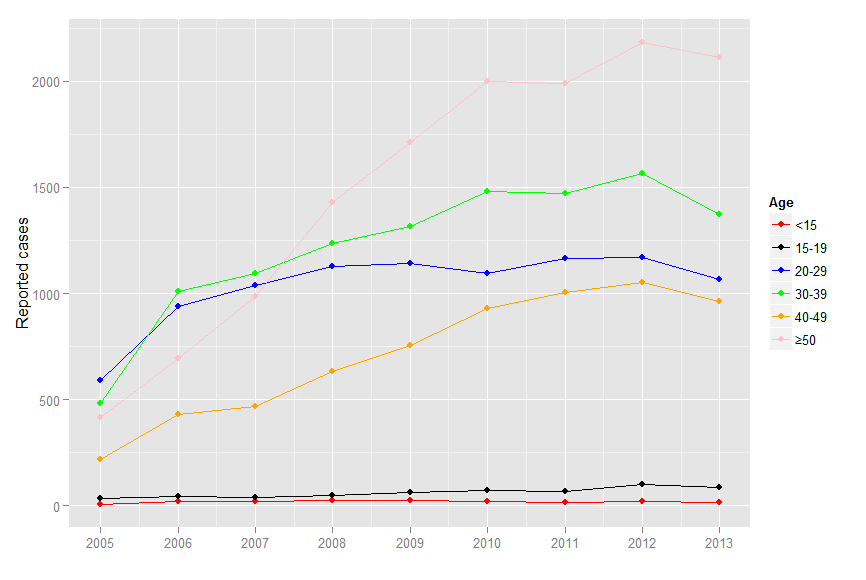

Supplement: Additional file 2: Figure S2. — Age-specific latent syphilis cases in Guangzhou from 2005 to 2013. (TIFF 1471 kb) [file 12879_2015_1072_MOESM2_ESM.tiff]
